# A Practitioner in Derbyshire, in Reply to Crito

**Published:** 1808-06

**Authors:** 


### *To the Editors of the Medical and Physical Journal.*

Gentlemen,

THE papers of Crito and those of a Practitioner in Derbyshire, will probably be thought by many, better calculated to amuse their respective writers, than to throw a fresh lustre on the pages of the Medical Journal. Though impressed with this fact, I am again induced to offer for your consideration a few observations arising out of the singular opinions promulgated by Crito in his last paper.—First noticing, by the way, the disposition which he manifests of forsaking the pale of his own argument, to tread on ground that must be perfectly strange to him: I mean, in his allusion and direct mention of a public dissection at Derby. The opinion, which I originally entertained, of an insulated part of this dissection, I now feel it necessary to say, remains unaltered; but as it has been already, perhaps, more than sufficiently canvassed in some of your late Numbers, I shall not dwell upon it here, farther than to ask, how Crito can connect this dissection with an argument on the most rational method of securing a bleeding artery? Could he think, by thus giving it fresh publicity, again to involve me with that memorable advo-

 cate, cate, Moromastix, and so work a diversion in his own favour?

We shall leave Crito in the undisturbed enjoyment of the early part of his paper, and shall commence with him at the "Old Nomenclature;" which indeed to us is perfectly new, being not aware that any distinct work of that kind, "on certain and rational principles," marking and regulating the many absurdities of anatomical expression, ever existed, till the late one of Dr. Barclay. Be this, however, as it may, we have not, it is hoped, greatly offended against the taste of the "Anatomical Connoisseur," (if any of your readers are inclined to assume so unnatural an appellation) in applying the epithet perverse, to that, which the greatest surgeons acknowledge to us, "often so perversely bleeds."

That Crito should adhere with such invincible pertinacity to his case of wounded ulnar artery, is not so much to be wondered at; for he who once establishes a position is seldom willing to abandon it. But is the practice which he pursued in it, such as we are bound to imitate or reject? This leads us to consider a strong question. "In a case where there was not the possibility of securing the artery, is it so surprising," says he, "to meet a recurrence of hæmorrhage?" Certainly, not at all surprising. But will it be argued, that such impossibility existed here? At least, let us first know, whether the previous and grand step towards security, ample dilatation, was ever had recourse to; as such a circumstance is never hinted at in the history of the case, we conclude it was not. It may be argued, perhaps, that an incision in parts so swollen and ill-conditioned, could be of no avail. That such a condition of the arm would make it infinitely more difficult to explore the artery, is beyond doubt; perhaps it might make its discovery even impossible; but, if we resolve upon doing something, there can be no question surely as to the choice of the means; that is, whether by free incision we would endeavour to expose the artery, or strike adventurously with the needle. It will be recollected, that we are speaking of the ulnar artery only, when wounded at the wrist; for that there may be cases of hæmorrhage from the trunks of arteries, or from peculiarity of situation, where to perform a deliberate operation would eminently risk, if not absolutely sacrifice, the life of the patient, is a position which will not be controverted; but such an argument does not hold here. We select the following quotation as peculiarly applicable to the case before us. "Suppose that a sura surgeon does not dissect neatly for the radial or ulnar artery at the wrist, but plunges for it with his needle, the skin, tendons, and nerves are included, dangerous nervous symptoms may ensue, or more certainly the next day, by the fading of the parts, the ligature slackens and the artery bleeds again."

We are sorry to hear of those secondary hæmorrhages so frequently happening after the operation of amputation; but while we regret the fact, we more than doubt the possibility of the solution of it, as offered by Crito. "From the suppuration loosening the ligatures too quickly." Crito ridicules the idea of attributing contraction of the fingers to an insulated or irritated state of the correspondent nerve by ligature, and is peculiarly happy in the supposed case which he gives us, illustrative of his idea of contraction. "Had the tendons of the wrist been cut," says he, "I believe the hand would have suffered contraction." He might positively have asserted it; but what is this, in the first instance, more than the natural action of muscles having lost their antagonist. Very unlike to that preternatural action, continued, and rigid from the first, hardly extensible by any counteracting force, which we have said, and it remains to be disproved, would happen, if the surgeon, plunging with the needle, includes the nerve as well as artery. A loss of feeling only, he apprehends, would be the consequence of such an accident; and, indeed, by a subsequent query, he would even make this effect doubtful; from which we infer, that he supposes as great a provision, by anastomosis, in the nervous, as is well known to exist in the arterial system. Opinions so contrary to fact and experience, meet in their bare mention, their own refutation.

It would seem, in short, as if Crito had not given this simple point of surgery his best consideration; and, perhaps, we cannot do better than to conclude in the words of a most excellent anatomist and surgeon, who, when addressing the student on this subject, says, "I would recommend it to him to read Mr. John Bell's Principles of Surgery upon this point, where he will find cases so pictured and represented to him, that he will not quickly forget them."

A PRACTITIONER IN DERBYSHIRE.

*March* 25, 1808.

